# The Diagnosis of Intrapulmonary Metastasis Multifocal Pulmonary Ground-Glass Nodules Based on Oncogenic Driver Mutation: Two Case Reports and Review of Literature

**DOI:** 10.3389/fsurg.2021.812559

**Published:** 2022-01-20

**Authors:** Yingkuan Liang, Kaiwen He, Biao Zhang, Ke Chen, Liangbin Pan, Guocai Mao, Yu Feng, Shaomu Chen, Haitao Ma

**Affiliations:** ^1^Department of Thoracic Surgery, The First Affiliated Hospital of Soochow University, Suzhou, China; ^2^Department of Thoracic Surgery, Suzhou Dushu Lake Hospital, Suzhou, China

**Keywords:** intrapulmonary metastasis, ground-glass nodules (GGNs), epidermal growth factor receptor mutation (EGFR mutation), HER2 mutations, multiple GGOs

## Abstract

With the increased use of low-dose computed tomography (LDCT), the detection of multifocal pulmonary ground-glass nodules (GGNs) has increased. According to the current clinical guidelines, multifocal GGNs tend to be treated as the multiple primary early-stage lung adenocarcinoma. However, studies have indicated that parts of multiple GGNs may originate from single nodules *via* intrapulmonary metastasis (IPM). Such IPM indicates the advanced stages even when the multiple GGNs are present as the early characteristics in pathological assessments. However, no gold standard exists for the differential diagnosis of multiple IPM GGNs. Here, we report two multifocal pulmonary GGNs cases where panel sequencing (672 driver mutation loci) showed that patient 1 (P1) shared two rare epidermal growth factor receptor (EGFR) mutations (primary L747_T751del and primary T790M) in the left upper lobe anterior segment and left lower lobe superior segment, respectively. Patient 2 (P2) shared a low-frequency human epidermal growth factor receptor 2 (HER2) mutation (primary Tyr772_Ala775dup) in two GGNs located in the apicoposterior and superior lingular segment of the left lower lobe (LLL). Oncogenic driver mutations were concordant between primary tumors and metastasis. Thus, shared low-frequency driver mutations in multiple GGNs strongly suggested that IPM existed with a high probability in these patients. Also, tumor cell spread through air spaces (STAS) was identified in pathological sections of the left upper lobe (LUL) nodule of P1, suggesting aerogenous spread may have been an effective pathway for IPM. Our report suggests that oncogenic driver mutations have prominent diagnostic value for IPM. Also, GGN IPM may occur in one lung lobe and between in different lung lobes.

## Introduction

Pulmonary ground-glass nodules (GGNs) are defined as hazy opaque growths that do not obscure the underlying bronchial structures or pulmonary vessels *via* a high-resolution computed tomography. The phenomenon was first indicated by the Nomenclature Committee of the Fleischner Society in 1996 (1). In the clinic, GGNs are normally classified as pure or mixed structures based on the inclusion of part-solid components (2). Usually, GGNs are present as benign lesions or relatively low-grade malignant lesions, such as the atypical adenomatous hyperplasia, adenocarcinoma *in situ*, or the minimally invasive adenocarcinoma (MIA) (3). While the 5-year survival rate of multiple GGNs is ~90%, a very small proportion of patients will present with poor outcomes (4), therefore, investigations are warranted.

In current clinical guidelines, multiple GGNs are considered multiple primary lung cancers (MPLCs) with minimal metastatic input (4). Genetic alterations in multiple GGNs are also considered effective clonality markers. Due to oncogenic driver mutation concordance between the primary lung cancer and the metastasis, several clinical studies have reported that MPLCs, including most multiple GGNs, are independently derived. Considerable genetic heterogeneity in different nodules is the most significant MPLC characteristic (5, 6). Currently, few studies have suggested that small proportions of multiple GGNs are derived from single clonality sites and are disseminated *via* intrapulmonary metastasis (IPM) based on similar low-frequency somatic mutations (7–9). The clonal relationship of multiple GGNs plays a critical role in the identification of IPM. Li et al. reported that a patient with five lesions shared a single mutation in the epidermal growth factor receptor (EGFR) (p.L858R), and the identification of a common origin strongly suggested IPM in multiple GGNs in a single lung lobe, even in pure GGNs (8). In other studies, oncogenic driver mutations, such as EGFR, *K-ras*, BRAF, PIK3CA, ALK, ROS1, RET, and HER2/TP53 in lung nodules, significantly improved the clonality assessments in patients with multiple lung nodules, including GGNs (9, 10).

In this study, patient 1 (P1) shared two rare primary EGFR mutations (T790M and L747_T751del) in two lesions from homolaterally two different lung lobes (left upper lobe anterior segment and left lower lobe superior segment). In another case, patient 2 (P2) shared a low-frequency primary human epidermal growth factor receptor 2 (HER2) (Tyr772_Ala775dup) mutation in two GGN lesions. Both were located in the left upper lobe (apicoposterior and superior lingular segments). No other oncogenic driver mutations were identified. Interestingly, tumor cells that were spread through air spaces (STAS) were identified in a pathological section from the left upper lobe nodule of P1. Based on shared rare/low-frequency mutations, we suggest these two multiple GGN cases may have exhibited IPM *via* an aerogenous spread. These findings highlight the differential diagnostic value of oncogenic driver mutations in multiple IPM GGNs, and they strongly suggest that an aerogenous spread was the IPM mechanism.

## Case Presentation

**Figure 1:**
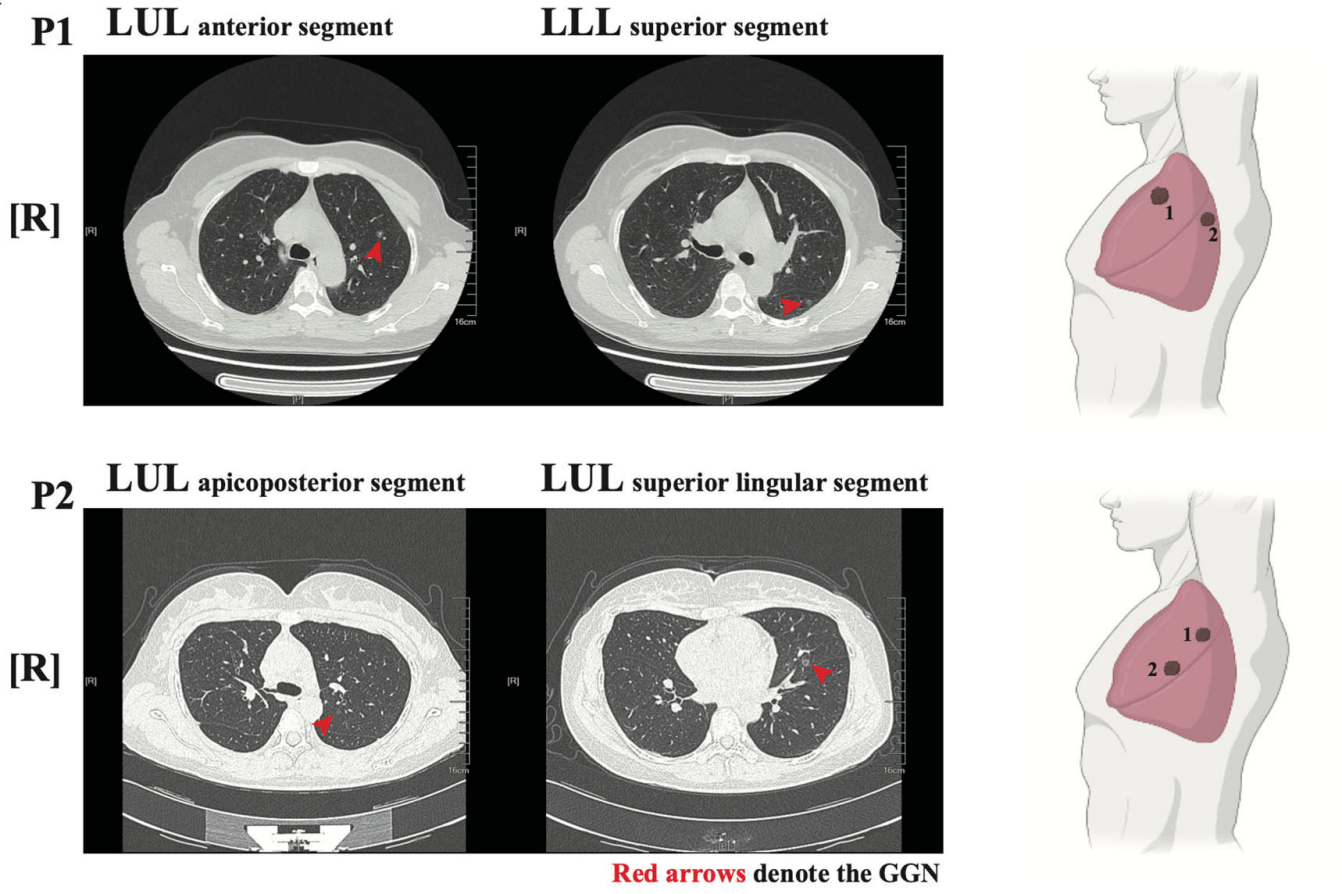
Radiological features of the two patients. left panel: Chest low-dose CT (LDCT) scans with two ground-glass nodules (GGNs, red arrows) of P1/P2; Right panel: illustration showed the location of GGNs in lung lobes.

Two female patients (P1 and P2) had no history of underlying disease, previous operations, or taking target medications (EGFR tyrosine kinase inhibitors, etc.). Both were non-smokers and in good general condition without any relevant symptoms. From a physical examination, two GGNs were identified in the left upper lobe (LUL) anterior segment and left lower lobe (LLL) superior segment of P1, who was 49 years old. Radiologically, the maximum diameter of both GGNs was 8.3 and 7.6 mm, respectively (Figure 1 upper panel). The bigger nodule (N1) had a 30% sub-solid component and the other was pure GGN.

For P2, who was 36 years old, both GGNs were located in the LUL (apicoposterior and superior lingular segments, respectively). The maximum diameters of both GGNs were 6.5 and 8.3 mm, respectively (Figure 1 lower panel). The bigger nodule (N2) had nearly a 60% sub-solid component while the smaller one was pure GGN.

**Figure 2:**
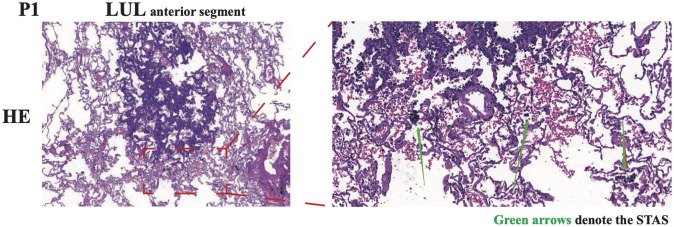
Representative images of HE immunohistochemical staining of N1 in P1. The spread through air spaces (STAS) of tumor cells was denoted by green arrows. LUL, left upper lobe.

After an antibiotic treatment cycle, the GGNs did not shrink. After further assessment, both patients were admitted for surgery, and in December 2020, both underwent wedge resection in four lesions. According to post-operative pathology, four lesions were diagnosed as lung MIA without lymphatic or distant metastasis (Table 1). Immunopathological results indicated the same pathological features and characteristics in both GGNs of P1: CK7 (+), TTF-1 (+), Napsin A (+), CK5/6 (–), P40 (–), P63 (+), ALK (D5F3) (–), Ki-67 (1%, +), and PD-L1 (22C3) (TPS < 1%). For P2, the immunopathological findings of both GGNs were CK7 (+), TTF-1 (+), Napsin A (+), CK5/6 (–), ALK (D5F3) (–), Ki-67 (3%, +), and PD-L1 (22C3) (TPS < 1%). Surprisingly, tumor cell STAS was identified in the N1 pathological section of P1 (Figure 2).

**Table 1:** Clinicopathological characteristics and mutation status of P1 and P2.

**Case**	**Sex**	**Age (y)**	**Nodules numbers**	**Location**	**Size (mm)**	**Radiological**	**Histology**	**Therapy**	**Mutation status-frequency**
P1	F	49	N1	LUL Anterior segment	8.3	Subsolid (30%)	MIA	WR	EGFR (p.T790M)-18.78% EGFR (p.L747_T751del)-11.57%
			N2	LLL Superior segment	7.6	pGGN	MIA	WR	EGFR (p.T790M)-1.87% EGFR (p.L747_T751del)-0.93%
P2	F	36	N1	LUL Apicoposterior	6.5	pGGN	MIA	WR	HER2 (p.Tyr772_Ala775dup)-2.7%
			N2	LUL Lingular segment	8.3	Subsolid (60%)	MIA	WR	HER2 (p.Tyr772_Ala775dup)-3.9%

*LUL, left upper lobe; LLL, left lower lobe; pGGN, pure pulmonary ground-glass nodule; MIA, minimally invasive adenocarcinoma; WR, wedge resection; EGFR, epidermal growth factor receptor; HER2, Human epidermal growth factor receptor 2*.

Panel sequencing (672 oncogenic driver mutation loci) was performed based on the four GGN lesions in the post-operative period in both patients. In P1, a shared rare mutation in EGFR (primary T790M) was identified with a mutant frequency of 18.78% in the LUL nodule (N1) and 1.87% in the LLL nodule (N2). Another low-frequency EGFR mutation [primary L747_T751del (exon 19 deletion)] was also detected in two nodules. The mutant frequencies were 11.57% in N1 and 0.93% in N2.

Additionally, in P2, a shared low-frequency mutation in HER2 (primary Tyr772_Ala775dup) was identified in both GGNs. The mutation frequencies were 2.7% in the apicoposterior segment and 3.9% in the superior lingular segment (Table 1). Peripheral blood was also analyzed in panel sequencing as a germline comparator and showed no similar mutations in the genetic background. Moreover, no other significant copy number variations, genetic rearrangements, or driver mutation fusions were identified in cancer-associate genes (e.g., K-ras, MET, RET, and ROS).

Based on this driver mutation data, we hypothesized that both GGNs in P1 or P2 had common clonal origins. In addition, STAS status in P1 suggested IPM may have occurred *via* an aerogenous spread.

## Discussion

Based on favorable outcomes and decreased propensity for nodal and distant metastasis, multiple GGNs are primarily recognized as an early-stage primary independent lung cancer. However, <10% of patients with multiple GGN have poor survival outcomes even though their nodules are classified as stage I. Thus, we speculated whether these patients were in early disease stages. Asmar et al. reported that the concordance rate of driver mutations (e.g., EGFR, KRAS, BRAF, and ALK) was up to 96% between primary lung cancer and metastasis (11). Thus, genetic heterogeneity is the most significant characteristic in MPLCs. Asmar also indicated that in their MPLC cohort, 40% of same-lobe nodules and 7.4% of multiple lobe nodules were IPM. Studies by Wu (12) and Chen (13) indicated that the clonality status of most multiple GGNs were wild with high driver mutation heterogeneity (> 80%) (12). Moreover, the identified GGNs in early tumorigenesis stages exhibited a less progressive accumulation of genetic alterations. Thus, multiple GGN lesions share similar driver mutations (rare/low-frequency mutations especially) and represent highly credible evidence for a common clonal original. According to the genetic results of P1, the two lesions in ipsilateral left lung upper and down lobes, respectively, shared a primary EGFR T790M mutation and a low-frequency EGFR [p. L747_T751del (exon 19 deletion)] mutation. The former mutation is typically recognized as a rare tyrosine kinase inhibitor (TKI) resistance mutation (0.5% in EGFR TKI-naïve patients with lung cancer). In addition, the L747_T751del EGFR mutation was also unusual; its incidence rate was 0.11% in AACR lung cancer cases (14). Research from Li et al. (15) reported that primary T790M EGFR mutations coexisted more frequently with L858R, but an acquired T790M mutation was more likely to coexist with exon 19 deletion. Thus, the combination of EGFR mutations (primary T790M and primary L747_T751del) was uncommon.

Similar to P1, P2 was identified with two GGN lesions in the same lung lobe and harbored a rare HER2 mutation (primary Tyr772_Ala775dup). The incidence of this subtype HER2 mutation is <1% in non-small cell lung cancers (14). Thus, the same underlying pathological features, e.g., similarities in rare gene mutations and no lymphatic/distant metastasis, led us to theorize that both lesions in P1 or P2 had originated *via* common clonality mechanisms. In addition, our genetic evidence suggested the possibility of multifocal IPM GGNs in the same lobe or, even, in differential lung lobes.

Aerogenous spread is a well-recognized and accepted tumor spread pattern in lung cancer. Numerous clinical, radiological, and pathological studies have suggested that an aerogenous spread is an independent risk factor and is related to the disease recurrence and surgical procedures. Aerogenous spread not only contributes to the same lobe aggressiveness, but also to ipsilateral lobe metastasis, ipsilateral lymph node metastasis, and even contralateral metastasis (16, 17). Based on our surgical, radiological, and pathological examinations, no lymphatic or hematogenous metastasis evidence was observed in our cases. A recent report on multiple GGNs considered that an aerogenous spread was the most likely pathway for IPM (8). Interestingly, tumor cell STAS was observed in a pathological section of the left upper lobe nodule of P1. Thus, our evidence supports these observations.

Distinct to the independent multifocal pulmonary GGNs, IPM GGNs are indicative of neoplastic nodules at advanced stages. In our cases, once an IPM GGN diagnosis was confirmed, the T1a would progress to the advanced T category (T4 for P1; T3 for P2). Hence, the segmentectomy and lobectomy might be a benefit for patients (16), and systemic chemotherapy and TKI therapy was necessary upon the surgery afterward. Thus, to distinguish the origin of multiple GGNs was with the extensive clinical value and application prospect. Currently,the diagnosis of multiple GGNs is primarily bases on imaging and pathological assessments, which was affected by the subjective of doctors. Thanks to the development of genetic sequencing technologies; oncogenic driver mutation analysis of clonal relationships in multiple GGNs may provide more accurate diagnoses. Clinical guidelines on multiple lung nodules have indicated that the identification of genetic features in multiple GGNs is valuable for a primary diagnosis or intrapulmonary spread (18, 19). Due to low morbidity and difficulty accessing tissues, we were unable to collect more cases to support our view. However, our research offers invaluable insights on the differential diagnosis of IPM GGNs.

In the clinic, patients with IPM GGNs may not benefit from limited resection. However, the gene sequencing could not be obtained before and during the surgery. The diagnosis of multiple GGNs and suitable procedural assessments is barely finished in the pre-operation. Therefore, efficient and fast detection methods to identify driver mutation status in multiple pulmonary GGNs require a future study.

## Data Availability Statement

The original contributions presented in the study are included in the article/supplementary material, further inquiries can be directed to the corresponding author/s.

## Ethics Statement

Written informed consent was obtained from the individual(s), and minor(s)' legal guardian/next of kin, for the publication of any potentially identifiable images or data included in this article.

## Author Contributions

YF and SC were the corresponding authors who identified this case. HM was the attending surgeon responsible for patients. YL, KH, and BZ were the resident surgeons and gathered the clinical information. KC, LP, and GM helped to write and edit the manuscript. All authors contributed to the article and approved the submitted version.

## Conflict of Interest

The authors declare that the research was conducted in the absence of any commercial or financial relationships that could be construed as a potential conflict of interest.

## Publisher's Note

All claims expressed in this article are solely those of the authors and do not necessarily represent those of their affiliated organizations, or those of the publisher, the editors and the reviewers. Any product that may be evaluated in this article, or claim that may be made by its manufacturer, is not guaranteed or endorsed by the publisher.
